# Dopamine Modulates Homeostatic Excitatory Synaptic Plasticity of Immature Dentate Granule Cells in Entorhino-Hippocampal Slice Cultures

**DOI:** 10.3389/fnmol.2018.00303

**Published:** 2018-08-30

**Authors:** Andreas Strehl, Christos Galanis, Tijana Radic, Stephan Wolfgang Schwarzacher, Thomas Deller, Andreas Vlachos

**Affiliations:** ^1^Neuroscience Center, Institute of Clinical Neuroanatomy, Goethe-University Frankfurt, Frankfurt, Germany; ^2^Department of Neuroanatomy, Institute of Anatomy and Cell Biology, Faculty of Medicine, University of Freiburg, Freiburg, Germany

**Keywords:** homeostasis, synaptic scaling, neurogenesis, hippocampus, neuromodulation, stem cells

## Abstract

Homeostatic plasticity mechanisms maintain neurons in a stable state. To what extent these mechanisms are relevant during the structural and functional maturation of neural tissue is poorly understood. To reveal developmental changes of a major homeostatic plasticity mechanism, i.e., homeostatic excitatory synaptic plasticity, we analyzed 1-week- and 4-week-old entorhino-hippocampal slice cultures and investigated the ability of immature and mature dentate granule cells (GCs) to express this form of plasticity. Our experiments demonstrate that immature GCs are capable of adjusting their excitatory synaptic strength in a compensatory manner at early postnatal stages, i.e., in 1-week-old preparations, as is the case for mature GCs. This ability of immature dentate GCs is absent in 4-week-old slice cultures. Further investigations into the signaling pathways reveal an important role of dopamine (DA), which prevents homeostatic synaptic up-scaling of immature GCs in young cultures, whereas it does not affect immature and mature GCs in 4-week-old preparations. Together, these results disclose the ability of immature GCs to express homeostatic synaptic plasticity during early postnatal development. They hint toward a novel role of dopaminergic signaling, which may gate activity-dependent changes of newly born neurons by blocking homeostasis.

## Introduction

Homeostatic plasticity plays a fundamental role in maintaining neural networks in a stable state. Among the best studied forms is homeostatic synaptic plasticity, which adjusts synaptic strength in a compensatory manner to changes in network activity (Davis, 2006; Marder and Goaillard, 2006; Turrigiano, 2008; Pozo and Goda, 2010). In recent years, numerous studies have addressed the cellular and molecular mechanisms of homeostatic synaptic plasticity in various experimental conditions (e.g., Marder and Goaillard, 2006; Turrigiano, 2012; Tien and Kerschensteiner, 2018). Yet, its relevance in neural development and maturation remains poorly understood. In this context, we hypothesized that homeostatic plasticity, i.e., negative feedback mechanisms that aim at stabilizing neural networks by preventing major changes in network structure and function, may hinder the efficient activity-dependent maturation and network integration of neurons. Indeed, in an earlier study we were able to provide evidence that CA1 pyramidal neurons acquire homeostatic properties between 2 weeks and 4 weeks of postnatal *in vitro* maturation (Vlachos et al., 2013), suggesting that homeostatic synaptic plasticity is a property that neurons acquire during the course of maturation, i.e., after a set-point is reached.

The dentate gyrus of the hippocampus is an attractive model to study homeostasis of immature neurons, since dentate granule cells (GCs) are born and differentiate postnatally, both *in vivo* (Altman and Das, 1965; van Praag et al., 2002; Toni et al., 2007; Kempermann et al., 2015; Nicola et al., 2015; Cahill et al., 2017; Radic et al., 2017a) and *in vitro* (Raineteau et al., 2004; Radic et al., 2017b). Previous work has demonstrated that immature GCs are capable of expressing Hebbian forms of synaptic plasticity, i.e., long-term potentiation (LTP) of excitatory synaptic strength (e.g., Schmidt-Hieber et al., 2004; Gonçalves et al., 2016). Hitherto, it has not been tested whether immature GCs also express homeostatic synaptic plasticity.

In this study entorhino-hippocampal slice cultures were treated with a well-established pharmacological protocol, i.e., network activity blockade with tetrodotoxin (TTX; 2 μM; for 3 days), to test for dentate GC excitatory synaptic up-scaling (Turrigiano et al., 1998). Compensatory changes in excitatory synaptic strength were assessed using whole-cell patch-clamp recordings of immature and mature GCs in young (5–6 days *in vitro*; 1-week-old) vs. old (22–25 days *in vitro*; 4-week-old) slice cultures. Considering that dopamine (DA) modulates the ability of GCs to express Hebbian plasticity (Mu et al., 2011), we sought to test for the relevance of DA in regulating homeostatic excitatory synaptic plasticity of dentate GCs.

## Materials and Methods

### Ethics Statement

All experimental procedures were performed according to the German animal welfare legislation and approved by the local animal welfare officers of Goethe-University Frankfurt and Albert-Ludwigs-University Freiburg, Faculties of Medicine. Mice were maintained in a 12 h light/dark cycle with food and water available *ad libitum*. Every effort was made to minimize distress and pain of animals.

### Preparation of Slice Cultures

Entorhino-hippocampal slice cultures were prepared at postnatal day 4–5 from C57BL/6J and CB6F1/J mice of either sex as previously described (Vlachos et al., 2012). Cultures were allowed to mature *in vitro* for either 5–6 days (1-week-old) or 22–25 days (4-week-old).

### Pharmacology

Slice cultures were treated with TTX (sodium-channel blocker; 2 μM; BioTrend), DA (20 μM; TOCRIS), SCH23390 hydrochloride (D_1_-like antagonist; 40 μM; TOCRIS), SKF81297 hydrobromide (D_1_-like agonist; 20 μM; TOCRIS), quinpirole hydrochloride (D_2_-like agonist; 20 μM; TOCRIS) for 3 days. Incubation medium was replaced once with fresh drug-containing medium.

### Whole-Cell Patch-Clamp Recordings

Whole-cell patch-clamp recordings from dentate GCs of slice cultures were carried out at 35^°^C as described previously (≤5 neurons per culture; Vlachos et al., 2012). The bath solution contained (in mM) 126 NaCl, 2.5 KCl, 26 NaHCO_3_, 1.25 NaH_2_PO_4_, 2 CaCl_2_, 2 MgCl_2_, 10 glucose. Patch pipettes contained (in mM) 126 K-gluconate, 10 HEPES, 4 KCl, 4 ATP-Mg, 0.3 GTP-Na_2_, 10 PO-Creatine and 0.3% (w/v) Biocytin (pH = 7.25 with KOH, 290 mOsm with sucrose) having a tip resistance of 4–6 MΩ. In some experiments Alexa488 or Alexa568 (both 10 μM) was added to the internal solution to visualize neuronal morphology prior to recordings. Neurons were recorded at holding potential of −70 mV in the presence of 10 μM D-APV and 0.5 μM TTX. In presence of the α-amino-3-hydroxy-5-methyl-4-isoxazolepropionic acid (AMPA) receptor antagonist CNQX (10 μM) no postsynaptic current events were detected (data not shown). Series resistance was monitored in 2 min intervals, and recordings were discarded if the series resistance and leak current changed significantly and/or reached ≥30 MΩ or ≥100 pA, respectively. Data were low pass filtered at 6 kHz and digitized at 10 kHz.

### Immunostaining and Imaging

Slice cultures were fixed, re-sliced and immunostained as described previously (Lenz et al., 2016) with antibodies against doublecortin (DCX; C-18; goat; Santa Cruz; 1:500) and calbindin (Calb; D28-K, mouse; Swant; 1:1,000). For *post hoc*-immunostainings, slice cultures were incubated with Alexa568-conjugated streptavidin (Life Technologies; 1:500). Confocal images were acquired using a Nikon Eclipse C1si laser-scanning microscope equipped with a 40× oil-immersion objective lens (NA 1.3; Nikon) and a 60× oil-immersion objective lens (NA 1.4; Nikon).

### Discrimination Between Mature and Immature Dentate Granule Cells

Recorded GCs were *ad hoc* characterized based on the position of the soma within the GC layer (GCL) and their resting membrane potential (RMP) and capacitance (C; Spampanato et al., 2012). GCs with a RMP > −65 mV and C <30 pF were considered immature. Some of the recorded cells were *post hoc* identified, and their developmental stage was assessed using immunostaining with antibodies against DCX and Calb (Duan et al., 2008).

### Quantification and Statistics

Immunostainings were analyzed using the ImageJ software package (Schindelin et al., 2012). To quantify the distribution of DCX- and Calb-positive GCs, confocal image stacks of the dentate gyrus were analyzed using the cell-counter plugin for ImageJ.

Electrophysiological data were filtered using a low pass elliptic filter with a cutoff frequency of 1 kHz and analyzed off-line by two independent observers using pClamp 10.2 (Axon Instruments, USA) and MiniAnalysis (Synaptosoft, Decatur, GA, USA). 150–300 miniature excitatory postsynaptic current (mEPSC) events were analyzed per recorded neuron.

Statistical comparisons were performed using non-parametric tests, i.e., Mann-Whitney test (to compare two groups) or the Kruskal-Wallis test followed by Dunn’s *post hoc* test (for multiple comparisons) using GraphPad Prism 7 (GraphPad Software Inc.). *p* values smaller 0.05 were considered a significant difference. In the text and figure values represent mean ± standard error of the mean (SEM). *p < 0.05, **p < 0.01, ***p < 0.001, and non-significant differences are indicated by “NS.” U-values are provided for significant results in the figure captions.

### Digital Illustrations

Confocal image stacks were stored as TIF files. Figures were prepared using Photoshop graphics software (Adobe, San Jose, CA, USA). Image brightness and contrast were adjusted.

## Results

### Immature Dentate Granule Cells in 1-Week-Old Slice Cultures Scale Their Excitatory Synapses

**Figure 1:**
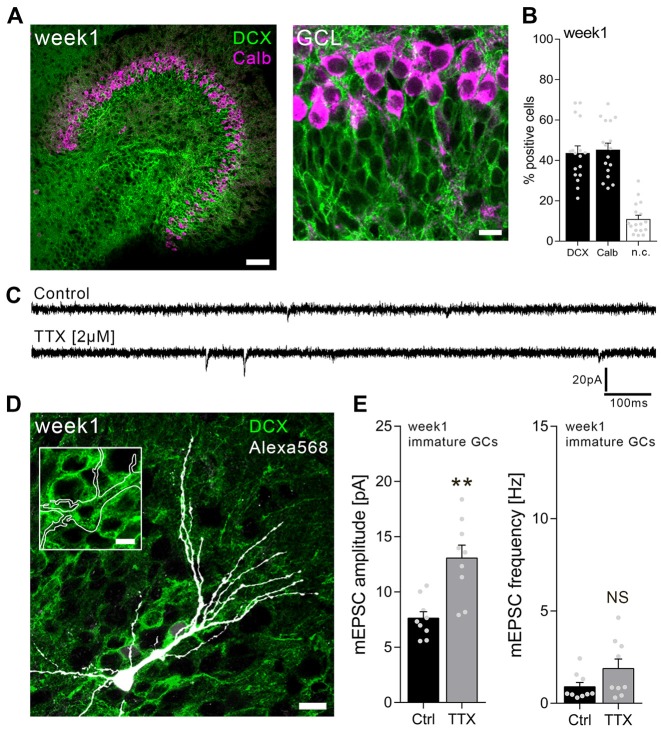
Immature dentate granule cells (GCs) of 1-week-old slice cultures express homeostatic excitatory synaptic plasticity. **(A)** Example of a 1-week-old entorhino-hippocampal slice culture stained for calbindin (Calb, magenta) and doublecortin (DCX, green). The GC layer (GCL) is shown at higher magnification. Scale bars, 50 μm and 10 μm. **(B)** Quantification of DCX and Calb expression in 1-week-old slice cultures. Some cells expressed both or neither of the markers (n.c., not classified; *n* = 17 cultures per group). **(C)** Sample traces of α-amino-3-hydroxy-5-methyl-4-isoxazolepropionic acid (AMPA) receptor-mediated miniature excitatory postsynaptic currents (mEPSCs) recorded from immature GCs in tetrodotoxin TTX (2 μM, 3 days) and vehicle-only (control)-treated cultures. **(D)** Example of a recorded (biocytin-filled), *post hoc* identified (streptavidin-Alexa568, white) immature GC in a DCX-stained slice culture. Scale bars, 10 μm and 5 μm (inset). **(E)** Group data of mEPSC recordings (amplitudes and frequencies) from immature GCs of vehicle-only-(Ctrl, control) and TTX-treated cultures (*n*_control_ = 9 cells, *n*_TTX_ = 9 cells; Mann-Whitney test; U-value = 6). Individual data points are indicated by gray dots. Values represent mean ± SEM (**p < 0.01; NS, non-significant difference).

Individual dentate GCs in the inner part of the GCL were patched in 1-week-old slice cultures, and AMPA receptor-mediated mEPSCs were recorded from TTX-treated cultures (2 μM; 3 days) as well as age-/time-matched vehicle-treated controls (Figure 1). Immature, i.e., DCX-positive, dentate GCs were readily detectable in 1-week-old slice preparations in this zone of the GCL (Figures 1A,B). In some cases, recorded dentate GCs were *post hoc* immunostained for DCX (Figure 1D), while position of the soma within the GCL and the RMP (RMP > −65 mV; mean: −46.4 ± 2.1 mV) served as primary criteria for immature GCs. It may be important to mention in this context that some cells exhibited a particular high RMP and did not show any mEPSC events, suggesting that AMPA receptor-carrying synapses were not yet formed on these cells (Espósito et al., 2005). mEPSCs were readily detectable in all neurons with RMP of −65 mV to −25 mV in this set of experiments (Figures 1C–E).

As shown in Figure 1E, a robust increase in excitatory synaptic strength was observed in immature GCs of TTX-treated slice cultures. Based on this result, we conclude that immature dentate GCs are capable of increasing their excitatory synaptic strength in a compensatory manner, similar to what is observed in mature cultured dentate GCs (Vlachos et al., 2012).

### Dopamine Blocks Homeostatic Plasticity in Immature but Not Mature Granule Cells of 1-Week-Old Cultures

**Figure 2:**
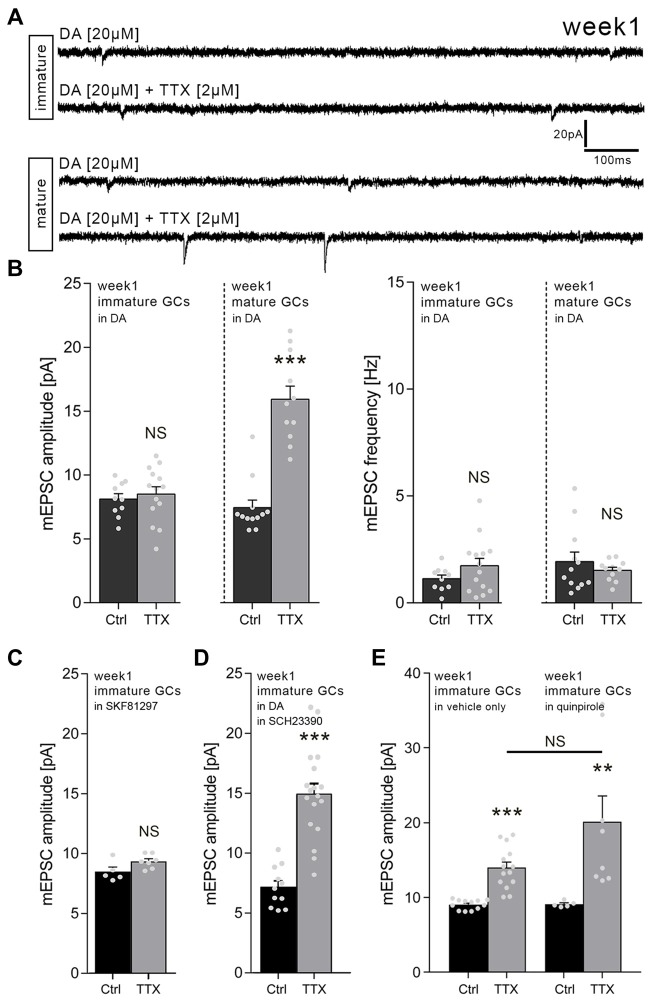
Dopamine (DA) prevents homeostatic synaptic plasticity of immature but not mature GCs in 1-week-old slice cultures. **(A)** Sample traces of AMPA receptor-mediated mEPSCs recorded from immature and mature GCs under various conditions (TTX, tetrodotoxin; DA, dopamine). **(B)** Group data of mEPSC recordings (amplitudes and frequencies). DA blocks TTX-induced excitatory synaptic scaling in immature but not mature GCs of 1-week-old slice cultures (immature: *n*_control_ = 10 cells, *n*_TTX_ = 14 cells; mature: *n*_control_ = 12 cells, *n*_TTX_ = 11 cells; Mann-Whitney test; U-value = 2). **(C)** The D_1_-like agonist SKF81297 (20 μM; 3 days) blocks TTX-induced excitatory synaptic scaling in immature GCs of 1-week-old slice cultures (*n*_control_ = 5 cells, *n*_TTX_ = 7 cells; Mann-Whitney test). **(D)** In presence of the D_1/5_ receptor antagonist SCH23390 (40 μM; 3 days) DA does not block TTX-induced excitatory synaptic scaling in immature GCs of 1-week-old slice cultures (*n*_control_ = 11 cells, *n*_TTX_ = 18 cells; Mann-Whitney test; U-value = 5). **(E)** The D_2_-like agonist quinpirole (20 μM; 3 days) does not affect TTX-induced excitatory synaptic scaling in immature GCs of 1-week-old slice cultures (vehicle-only: *n*_control_ = 12 cells, *n*_TTX_ = 14 cells; quinpirole: *n*_control_ = 5 cells, *n*_TTX_ = 8 cells; Kruskal-Wallis test followed by Dunn’s *post hoc* test). Individual data points are indicated by gray dots. Values represent mean ± SEM (**p < 0.01; ***p < 0.001; NS, non-significant difference).

In order to learn more about the signals that control homeostatic synaptic plasticity in immature dentate GCs, we tested for the role of DA. We speculated that signals which promote Hebbian plasticity may hinder the ability of immature GCs to express homeostatic synaptic plasticity, thus favoring their activity-dependent maturation. Hence, TTX-experiments were repeated in a different set of 1-week-old slice cultures which were treated with DA (20 μM; 3 days). Indeed, in the presence of DA, the TTX-induced increase in excitatory synaptic strength did not emerge in immature dentate GCs of 1-week-old cultures (Figures 2A,B).

Interestingly, DA did not affect TTX-induced synaptic up-scaling of mature, i.e., Calb-positive GCs (RMP ≤ −65 mV), which were recorded in the same set of cultures in these experiments (Figures 2A,B). We conclude that DA prevents homeostatic synaptic plasticity specifically in immature but not mature dentate GCs in 1-week-old slice cultures.

### D_1/5_ Receptor Activation Blocks Homeostatic Plasticity in Immature Granule Cells of 1-Week-Old Cultures

To confirm and extend these findings, TTX-experiments were repeated in a different set of 1-week-old slice cultures using the D_1/5_ receptor agonist SKF81297. Immature GCs did not increase their mEPSC amplitudes when TTX (2 μM) was applied together with 20 μM SKF81297 for 3 days (Figure 2C). TTX-induced synaptic scaling was not affected in presence of SKF81297 in mature dentate GCs recorded from the same set of cultures, while higher baseline mEPSC amplitudes were observed for mature GCs in these recordings (control: 17.9 ± 0.9 pA; TTX: 27.2 ± 2.1 pA; *n*_control_ = 8 neurons, *n*_TTX_ = 6 neurons; Mann-Whitney test, *p* < 0.001, U-value = 0). Consistent with this result, TTX-induced synaptic scaling of immature GCs emerged in presence of DA (20 μM), when the D_1/5_ receptor antagonist SCH23390 (40 μM) was co-applied (Figure 2D). We conclude that DA acts via D_1/5_ receptors to block TTX-induced homeostatic synaptic plasticity in immature GCs of 1-week-old cultures.

### The D_2_-Like Receptor Agonist Quinpirole Does Not Affect TTX-Induced Homeostatic Plasticity in Immature Granule Cells of 1-Week-Old Slice Cultures

The experiments in which we combined DA and the D_1/5_ receptor blocker SCH23390 argued against the involvement of other DA receptors, since these receptors are still activated by DA in the presence of SCH23390. Yet, we decided to err on the side of caution and hence tested for the effects of the D_2_-like agonist quinpirole. As shown in Figure 2E, TTX-induced synaptic scaling was not blocked in the presence of 20 μM quinpirole. Although a trend toward higher mEPSC amplitudes in the TTX + quinpirole group was detected, this effect did not reach the level of significance when compared to the TTX + vehicle only group (Figure 2E).

Taken together, these results confirmed once more that immature GCs of 1-week-old slice culture are capable of adjusting their excitatory synaptic strength in a compensatory manner. DA - likely acting via D_1/5_ receptors rather than D_2_-like receptors - blocks this property of immature GCs without affecting TTX-induced synaptic scaling in neighboring mature GCs.

### Homeostatic Synaptic Up-Scaling Is Not Observed in Immature Granule Cells of 4-Week-Old Cultures

**Figure 3:**
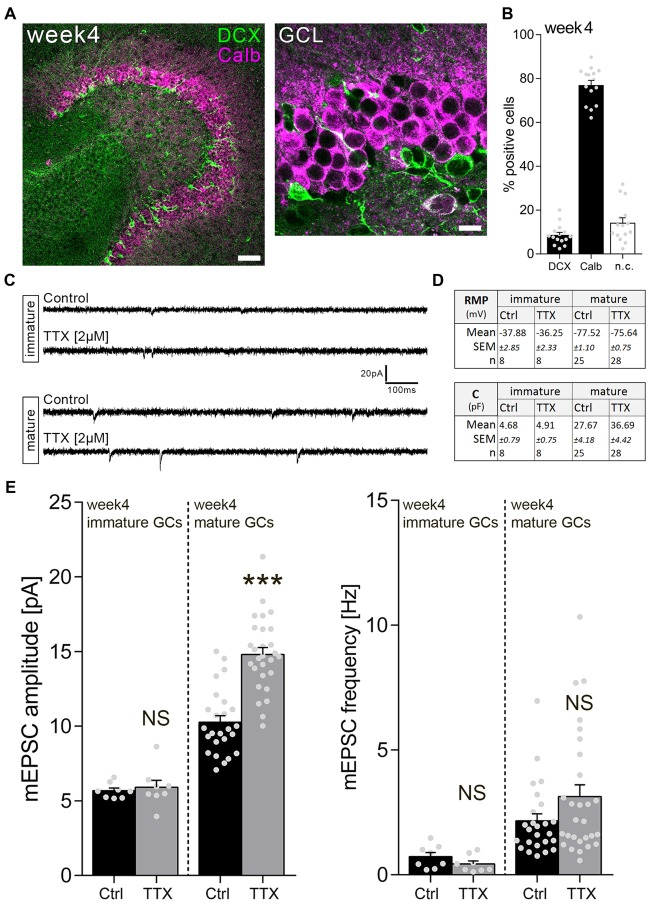
Immature dentate GCs of 4-week-old slice cultures do not express homeostatic excitatory synaptic plasticity. **(A)** Example of a 4-week-old entorhino-hippocampal slice culture stained for Calb (magenta) and DCX (green). The GCL is shown at higher magnification. Scale bars, 50 μm and 10 μm. **(B)** Quantification of DCX and Calb expression in 4-week-old slice cultures. Some cells expressed both or neither of the markers (n.c., not classified; *n* = 15 cultures per group). **(C)** Sample traces of AMPA receptor-mediated mEPSCs recorded from GCs in TTX (2 μM, 3 days) and vehicle-only (control)-treated cultures. **(D)** Resting membrane potential (RMP) and capacitance of immature and mature GCs were not changed after TTX treatment (Mann-Whitney test). **(E)** Group data of mEPSC recordings (amplitudes and frequencies) from immature and mature GCs of vehicle-only (Ctrl, control) and TTX-treated cultures (immature: *n*_control_ = 8 cells, *n*_TTX_ = 8 cells; mature: *n*_control_ = 25 cells, *n*_TTX_ = 28 cells; Mann-Whitney test; U-value = 57). Individual data points are indicated by gray dots. Values represent mean ± SEM (***p < 0.001; NS, non-significant difference).

We next wondered whether immature GCs in a more mature environment, i.e., 4-week-old slice cultures, behave similarly. Immunostainings revealed that the majority (about 80%) of GCs are Calb-positive in 4-week-old cultures (Figures 3A,B). Nevertheless, DCX-positive neurons are readily detectable in the more mature cultures, and these immature neurons disclose a significantly higher RMP compared to their mature GC neighbors (Figure 3D).

Surprisingly, in the light of our observations in 1-week-old cultures, TTX treatment (2 μM; 3 days) did not change mEPSC amplitudes of immature GCs, while inducing a robust homeostatic synaptic up-scaling response in mature GCs recorded in the same set of cultures (Figures 3C,E). Based on these results we conclude that immature GCs do not scale their excitatory synaptic strength in 4-week-old slice cultures.

### Dopamine Does Not Affect the Ability of Granule Cells in 4-Week-Old Cultures to Express TTX-Induced Synaptic Scaling

**Figure 4:**
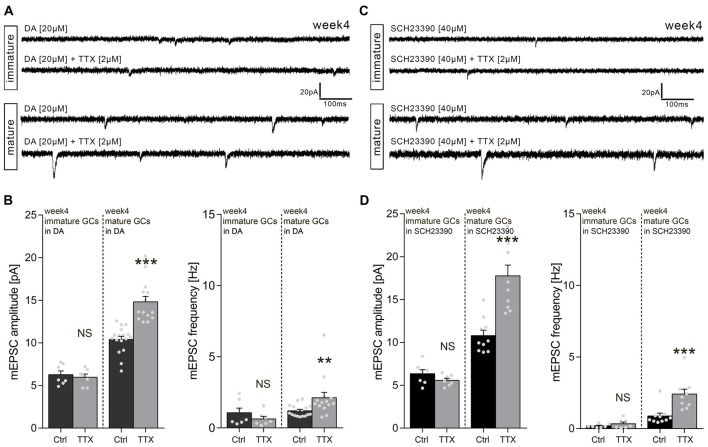
Dopamine (DA) has no effect on homeostatic excitatory synaptic plasticity of GCs in 4-week-old slice cultures. **(A)** Sample traces of AMPA receptor-mediated mEPSCs recorded from immature and mature GCs of 4-week-old entorhino-hippocampal slice cultures in presence of DA-only or DA + TTX. **(B)** Group data of mEPSC recordings (amplitudes and frequencies) from immature and mature GCs of 4-week-old slice cultures cultures (immature: *n*_control_ = 7 cells, *n*_TTX_ = 7 cells; mature: *n*_control_ = 17 cells, *n*_TTX_ = 14 cells; Mann-Whitney test; U-values ≤49.5). **(C)** Sample traces of AMPA receptor-mediated mEPSC recorded from immature and mature GCs of 4-week-old entorhino-hippocampal slice cultures in presence of SCH23390 (D_1/5_ receptor antagonist) or SCH23390 + TTX. **(D)** Group data of mEPSC recordings (amplitudes and frequencies) from immature and mature GCs of 4-week-old slice cultures cultures (immature: *n*_control_ = 7 cells, *n*_TTX_ = 7 cells; mature: *n*_control_ = 10 cells, *n*_TTX_ = 10 cells; Mann-Whitney test; U-values ≤6). Individual data points are indicated by gray dots. Values represent mean ± SEM (**p < 0.01; ***p < 0.001; NS, non-significant difference).

What is the effect of DA on TTX-induced synaptic scaling in 4-week-old slice cultures? To address this question, we treated a different set of 4-week-old cultures with TTX (2 μM) and DA (20 μM) for 3 days and recorded mEPSCs from immature and mature GCs within the same set of 4-week-old cultures (see Figure 2B). As shown in Figures 4A,B, DA treatment had no apparent influence on the effects of TTX: Immature GCs did not scale their excitatory synapses, while mature GCs exhibited a full-blown synaptic scaling response (thus confirming our previous findings in the absence of DA).

Finally, we tested for the possibility that an endogenous, i.e., hippocampal source of DA may have blocked the ability of immature GCs to scale up their excitatory synapses in response to TTX. Therefore, 4-week-old slice cultures were treated with TTX and the D_1/5_ receptor antagonist SCH23390 (Figures 4C,D), which rescued the scaling response of immature GCs in 1-week-old cultures in the presence of DA (see Figure 2D). Again, under these experimental conditions immature GCs did not scale their excitatory synapses, while a robust increase in mEPSC amplitudes was detected in mature GCs. No significant difference was detected by statistically comparing mEPSC amplitudes of mature GCs treated with TTX + SCH23390 and mature GCs treated with TTX-only (see Figure 3E). Taken together, we conclude that neither scaling of immature nor scaling of mature GCs is influenced by DA in 4-week-old slice cultures.

## Discussion

The relevance of homeostatic plasticity during neural development remains a matter of debate. Considering its stabilizing nature, it is conceivable that homeostasis hampers the ability of new neurons to integrate into networks, e.g., by counteracting major structural and functional changes such as dendritic or axonal growth and associative synaptic plasticity. Consistent with the hypothesis that homeostatic synaptic plasticity aims at keeping neurons in their current working range (Turrigiano, 2008, 2012), mature dentate GCs show a robust synaptic scaling response in 4-week-old entorhino-hippocampal cultures, while in the same set of cultures immature GCs do not adjust their excitatory synaptic strength in a compensatory manner. This situation may facilitate network integration of immature neurons, and after a set-point has been reached, homeostatic mechanisms emerge to stabilize the neurons in their mature state (see Vlachos et al., 2013).

The situation might be different during early postnatal development. In 1-week-old slice cultures immature GCs increase their excitatory synaptic strength in response to TTX treatment. To the best of our knowledge, this is the first description of an early homeostatic synaptic scaling response of immature neurons in an emerging organotypic network. What could be the relevance of such a mechanism? At this point we speculate that homeostatic mechanisms may keep immature neurons in a “ready and set” position, while regulatory signals, which suppress homeostasis, may provide the “go” signal for coordinated associative structural and functional changes.

DA is an interesting candidate molecule in this context. Evidence has been provided that DA mediates dendritic/axonal growth (Parish et al., 2001; Penrod et al., 2015; Shinohara et al., 2017) and facilitates the induction of Hebbian synaptic plasticity and learning and memory (Gangarossa and Valjent, 2012; Sariñana et al., 2014; Ntamati and Lüscher, 2016). Whether DA is instructive in mediating these effects, or rather permissive (by blocking homeostasis), requires further investigation.

Interestingly, in our experiments, DA does not affect the ability of mature GCs to express TTX-induced homeostatic excitatory synaptic scaling. Both in 1-week- and 4-week-old cultures DA has no apparent effect on baseline synaptic transmission and synaptic strengthening after TTX treatment, which may be one of the reasons why DA has not yet been linked to homeostatic synaptic plasticity. However, in immature GCs of 1-week-old slice cultures, DA blocks the ability of immature neurons to express homeostatic synaptic plasticity. Because pharmacological activation of D_1/5_ receptors (but not D_2/3_ receptor activation) mimics the effects of DA, and based on our experiments in which we used SCH23390 to block D_1/5_ receptors in presence of DA, we conclude that DA acts in a D_1/5_ receptor-dependent manner in our experimental setting. Hence, DA signaling via D_1/5_ receptors may act as a molecular switch that prevents homeostatic synaptic plasticity of immature neurons, which in turn may support their activity-dependent maturation during early postnatal development.

A recent study reported that early postnatal but not late adult neurogenesis is impaired in an animal model of Parkinson’s disease (Brandt et al., 2017). This observation is consistent with our *in vitro* experiments which show an influence of DA on immature neurons in young but not older slice cultures. Whether the lack of DA during early postnatal development and hence the inability to prevent homeostatic synaptic plasticity also affect neurogenesis remains to be shown. More work is required to learn about the interplay between homeostatic synaptic plasticity and neurogenesis/cell survival.

Why are immature GCs in 4-week-old slice cultures not adjusting their excitatory synaptic strength in a compensatory manner? In the experiments in which we used SCH23390 in 4-week-old slice cultures, we sought to test whether an endogenous source of DA exists in mature slice preparations which blocks homeostatic synaptic plasticity of immature GCs. Because synaptic scaling was not detected in the presence of the D_1/5_ receptor antagonist, we conclude that DA, i.e., the mechanism that blocks synaptic scaling in 1-week-old slice cultures, is not the major reason for the absence of synaptic scaling in immature GCs of 4-week-old slice cultures. It should be noted in this context that SCH23390 may also modulate serotonin receptors and inwardly rectifying potassium channels. Hence, although SCH23390 had no effect in 4-week-old cultures, it is interesting to hypothesize that other (local) factors may exist, which prevent homeostasis of immature neurons in more mature networks. Therefore, the state of maturation of the environment and specific factors secreted by neighboring neurons and glia cells may affect the ability of immature neurons to express this form of plasticity.

Whereas work from the past years has mainly focused on the mechanisms that mediate homeostasis, the results of the present study emphasize the relevance of signaling pathways that prevent homeostasis. DA qualifies as one of the factors that suppresses homeostasis specifically in immature neurons during early postnatal development. Hence, specific factors may exist which set the balance between homeostatic and Hebbian synaptic plasticity, affecting specific populations of neurons depending on their maturation state and the state of the network (for a recent review on the interplay between homeostatic and Hebbian plasticity, see Keck et al., 2017). Apparently, a better understanding of these mechanisms will provide new important insight on the role of homeostatic plasticity during development and network maturation, which may also be of relevance in the context of stem cell-based therapies and developmental brain diseases.

## Author Contributions

The study was conceived and supervised by AV. Experiments were designed by AS, CG, TD and AV. AS and CG performed experiments and analyzed the data with the help of TR. The manuscript was written by AV with the help of AS, CG, SS and TD. All authors were involved in data interpretation and critically revising the manuscript.

## Conflict of Interest Statement

The authors declare that the research was conducted in the absence of any commercial or financial relationships that could be construed as a potential conflict of interest.
